# Massively Parallel In Vitro Functional Analysis of Evolution-Derived Transcriptional Riboswitch Sequences

**DOI:** 10.21769/BioProtoc.5804

**Published:** 2026-09-05

**Authors:** Laura M. Hertz, Julius B. Lucks

**Affiliations:** 1 Interdisciplinary Biological Sciences Graduate Program, Northwestern University, Evanston, IL, USA; 2 Department of Chemical and Biological Engineering, Northwestern University, Evanston, IL, USA; 3 Center for Synthetic Biology, Northwestern University, Evanston, IL, USA

**Keywords:** Riboswitches, Intrinsic termination, In vitro transcription, RNA-seq, NGS, Bioinformatics

## Abstract

Riboswitches are structured non-coding RNA elements that regulate gene expression in response to small molecules; they serve as valuable systems in both public health and biophysical research by elucidating principles around RNA–ligand interactions, structure, and cellular function. Traditional approaches to studying riboswitches have relied on low-throughput techniques such as reporter assays or gel electrophoresis analysis of transcriptional products, which are limited in scalability. In this study, we present a high-throughput protocol to characterize the transcriptional activity of nearly 2,000 natural variants of the fluoride riboswitch in in vitro transcription. Starting with bioinformatics, we compiled a comprehensive dataset of riboswitch variants and then employed massive parallel oligonucleotide synthesis to generate an oligo pool of the riboswitch library. This pool was transcribed in vitro, converted into an Illumina-compatible next-generation sequencing (NGS) library, and analyzed to identify transcriptionally active riboswitch candidates. The workflow integrates natural riboswitch bioinformatic acquisition into a quantitative readout in a single streamlined pipeline, enabling large-scale exploration of transcriptional riboswitch function. This protocol offers a scalable method for mapping genotype-to-function relationships across transcriptional riboswitch families, accelerating the identification of functional variants for desired applications.

Key features

• Bioinformatics to acquire full sequences (aptamer + downstream expression platform) of naturally occurring riboswitches.

• *E. coli* RNA polymerase in vitro transcription to characterize nearly 2,000 fluoride riboswitches and analysis with NGS.

## Graphical overview


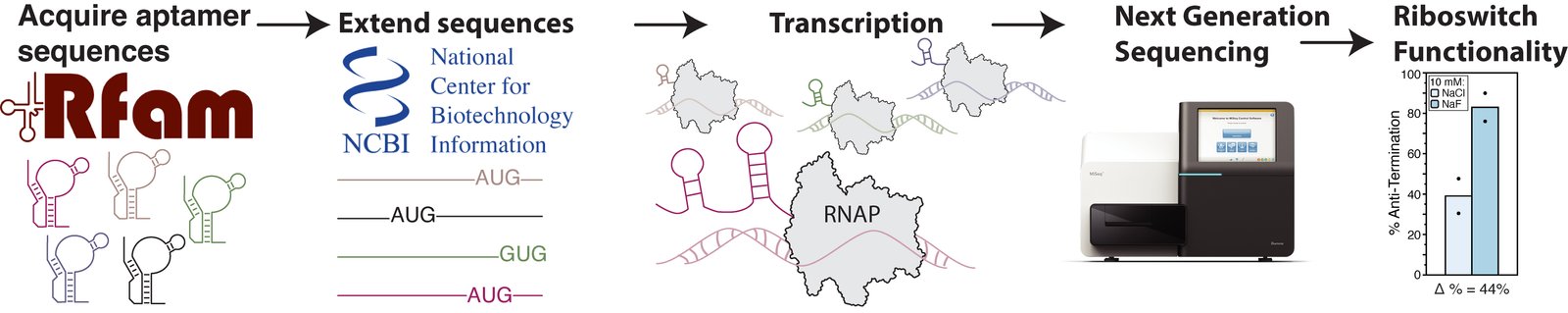


**Assessing the riboswitch functionality in an in vitro–transcribed context of known aptamers.** This figure is adapted from Hertz et al. (2026) *NAR* [1].

## Background

Riboswitches are important non-coding regulatory elements found across all kingdoms of life that have broad applications in public health [2] and as biophysical models [3]. Riboswitches contain two structural and functional domains: the aptamer domain that binds a ligand, and the expression platform that carries out the gene regulation mechanism. Structurally, the aptamer domains of riboswitches are highly evolutionarily conserved in order to carry out the singular role of ligand binding. Expression platforms are generally poorly conserved, as there are examples that can carry out different forms of gene regulation, including at the transcriptional [4], translational [5], degradation [6], and splicing levels [7]. As a result, there is relatively poor characterization of the function of all known riboswitches within a particular aptamer class. To fully explore riboswitches as genetic control elements in mechanistic and molecular tool development, we need high-throughput methods to characterize expression platform function.

Next-generation sequencing (NGS) is a powerful high-throughput method that can be applied to quantify riboswitch regulatory function. At the cellular level, RNA-seq data validates active riboswitches [8]. In synthetic systems, NGS can reveal optimal sequences to generate novel transcriptional riboswitches (histamine, tetR, etc.) or reveal translational sequence dependence [9,10]. Similarly, researchers characterized the self-cleavage activity of 2,625 natural Twister ribozymes [11]. We sought to characterize the computationally predicted fluoride aptamer sequences deposited on Rfam [12] as full fluoride riboswitches in a synthetic transcription environment to identify transcriptionally active variants. We employed bioinformatics to compile the riboswitch sequences and massively parallel oligo synthesis to generate the pool of riboswitch variants. Then, we performed transcription, generated an Illumina NGS library, and analyzed the output reads to show that this high-throughput pipeline is fit for identifying high-performing transcriptional riboswitch candidates.

## Materials and reagents

**Biological materials**

1. Oligo pool (Twist Biosciences)

2. Oligo sequences (Integrated DNA technologies, IDT)

**Table:** 

Name	Sequence	Supplier	Order specifications
Primer A	gcttccggcttgattctaaagatc	IDT	PAGE purified
Primer B	cggacagaaaatttgtgccc	IDT	PAGE purified
Linker	/5Phos/rCrUrGrArCrUrCrGrGrGrCrArCrCrArArGrGrA/3ddC/	IDT	Standard desalting
Primer C	/5BiosG/GTCCTTGGTGCCCGAGT	IDT	Standard desalting
SS2.0 Dumbbell	/5Phos/TGAAGAGCCTAGTCGCTGTTCANNNNNNCTGCCCATAGAG/3SpC3/	IDT	PAGE purified
Illumina INDEX Primers (Illumina Knowledge Article #7129)	CAAGCAGAAGACGGCATACGAGAT[INDEX]GTGACTGGAGTTCAGACGTGTGCTCTTCCGATCTTGAACAGCGAC TAGGCTCTTCA	IDT	PAGE purified
Primer D	CTTTCCCTACACGACGCTCTTCCGATCTYYYRGTCCTT GGTGCCCGAG*T*C*A*G	IDT	Standard desalting
TruSeq universal adapter	AATGATACGGCGACCACCGAGATCTACACTCTTTCCCTACACGACGCTCTTCCGATCT	IDT	Standard desalting

**Reagents**

1. RNase/DNase-free water (Invitrogen, catalog number: 10977-015)

2. dNTPs (NEB, catalog number: N0447L)

3. 5× Q5 reaction buffer (NEB, catalog number: B9027S)

4. Q5 polymerase (NEB, catalog number: M0491L)

5. Bead Purification kit (Cytiva, catalog number: 29343052)

6. Tris (Sigma, catalog number: T3253-500G)

7. EDTA pH 8.0 (Invitrogen, catalog number: 15575020)

8. KCl (Sigma-Aldrich, catalog number: P9541-500G)

9. DTT (Invitrogen, catalog number: 18090010); create 10-μL single-use aliquots and store at -20 °C

10. MgCl_2_ (NEB, catalog number: B0510A)

11. BSA (NEB, catalog number: B9000S)

12. *E. coli* RNAP holoenzyme (NEB, catalog number: M0551S)

13. NTPs (Fisher Scientific, catalog number: FERR1481)

14. Rifampicin (Sigma-Aldrich, catalog number: R3501-250MG)

15. NaCl (Sigma-Aldrich, catalog number: S3014-1KG)

16. TRIzol (Ambion, catalog number: 15596018)

17. Chloroform (Acros Organics, catalog number: 423555000)

18. GlycoBlue (Invitrogen, catalog number: AM9515)

19. Isopropanol (Sigma-Aldrich, catalog number: 190764-1L)

20. Ethanol (Sigma-Aldrich, catalog number: 459844-500ML)

21. 10× TURBO DNase buffer (Invitrogen, catalog number: 4022G)

22. TURBO DNase (Invitrogen, catalog number: AM2238)

23. 50% PEG800 (NEB, catalog number: 1004S)

24. 5′ Adenylation kit (NEB, catalog number: E2610S)

25. 10× T4 RNA ligase buffer and T4 RNA ligase, Trunc KQ (NEB, catalog number: M0373L)

26. SUPERase·In^TM^ RNase inhibitor (Invitrogen, catalog number: AM2696)

27. NaOAc (Invitrogen, catalog number: AM9740)

28. SSIV buffer and SSIV enzyme (Invitrogen, catalog number: 18090010)

29. NaOH (Sigma, catalog number: S5881)

30. HCl (Sigma, catalog number: 320331-500ML)

31. Betaine (Sigma, catalog number: B0300-1Vl)

32. T4 DNA ligase buffer and ligase enzyme (NEB, catalog number: B0202S)

33. ExoI (NEB, catalog number: M0293S)

34. High-sensitivity Qubit assay (Invitrogen, catalog number: Q32854)

35. DNA spin columns (EconoSpin, catalog number: 1910-250)

36. Buffer PB (Qiagen, catalog number: 19066)

37. Buffer PE (Qiagen, catalog number: 19065)

**Solutions**

1. 10× transcription buffer (see Recipes)

2. Transcription start/stop solution (see Recipes)

**Recipes**

**1. 10× transcription buffer**

**Table:** 

Reagent	Final concentration	Volume
RNase/DNase-free water	n/a	448 μL
Tris pH 8.0	200 mM	200 μL
EDTA pH 8.0	1 mM	2 μL
KCl	500 mM	250 μL
DTT (fresh or a one-use frozen aliquot)	10 mM	100 μL
Total	n/a	1 mL

*Note: For convenience, create a stock solution of the 10× TB* ***without DTT*** *and store at room temperature. During setup for the transcription reaction, prepare a fresh DTT solution or thaw a one-use aliquot (stored at -20 °C).*

**2. Transcription start/stop solution**

**Table:** 

Reagent	Final concentration	Volume
RNase/DNase-free water	n/a	15 μL
NTPs	5 mM	4 μL
Rifampicin	0.1 mg/mL	1 μL
Total	n/a	20 μL

*Note: Rifampicin is for performing a single round of transcription [13]. If multiple rounds of transcription initiation to have multiple RNA copies from one DNA template are desired, then replace rifampicin with water.*

**Laboratory supplies**

1. 1.7 mL tubes (Corning, catalog number: MCT-175-C)

2. 0.7 mL tubes (Bio Plas, catalog number: 4040)

3. PCR tubes (Eppendorf, catalog number: 951-01-002-2)

4. Magnetic stand for bead purification (Ergi Lab Supplies, catalog number: 1008)

5. QIAquick PCR Purification kit (50) (Qiagen, catalog number: 28104)

6. 20 μL pipette tips (Rainin, catalog number: 30389226)

7. 200 μL pipette tips (Rainin, catalog number: 30389239)

8. 1,000 μL pipette tips (Rainin, catalog number: 30389212)

## Equipment

1. Thermocycler (Bio-Rad, model: S1000^TM^)

2. Benchtop centrifuge (Eppendorf, model: EP5405000441)

3. Bioanalyzer (Agilent, model: 2100 bioanalyzer)

4. Freezer (-20 °C) (Thermo Scientific, model: MF02PA-SAEE-TS)

5. Qubit (Thermo Scientific, catalog number: Q33239)

6. Pipettes 2, 20, 200, 1,000 μL (Rainin, catalog number: 30579367)

7. Sequencing gel apparatus (Bio-Rad, catalog number: 165-3860)

## Software and datasets

**Table:** 

Type	Software/dataset/resource	Version	Date	License	Access
Code	GitHub, Code S1	0.0	Jan 2026	GNU v3.0	Free [14]
Data	Rfam	15.0	Sep 2024	CC0	Free [12]
Software	BacTermFinder	1.0	Dec 2024	GPL-3.0	Free [15]
Software	PEAR	0.9.6	Mar 2014	CCPL	Free [16]
Software	megahit	1.0.6.1	Jun 2017	GPL-3.0	Free [17]
Software	STAR	2.7.9a	May 2021	MIT	Free [18]

## Procedure

**Figure 1.**
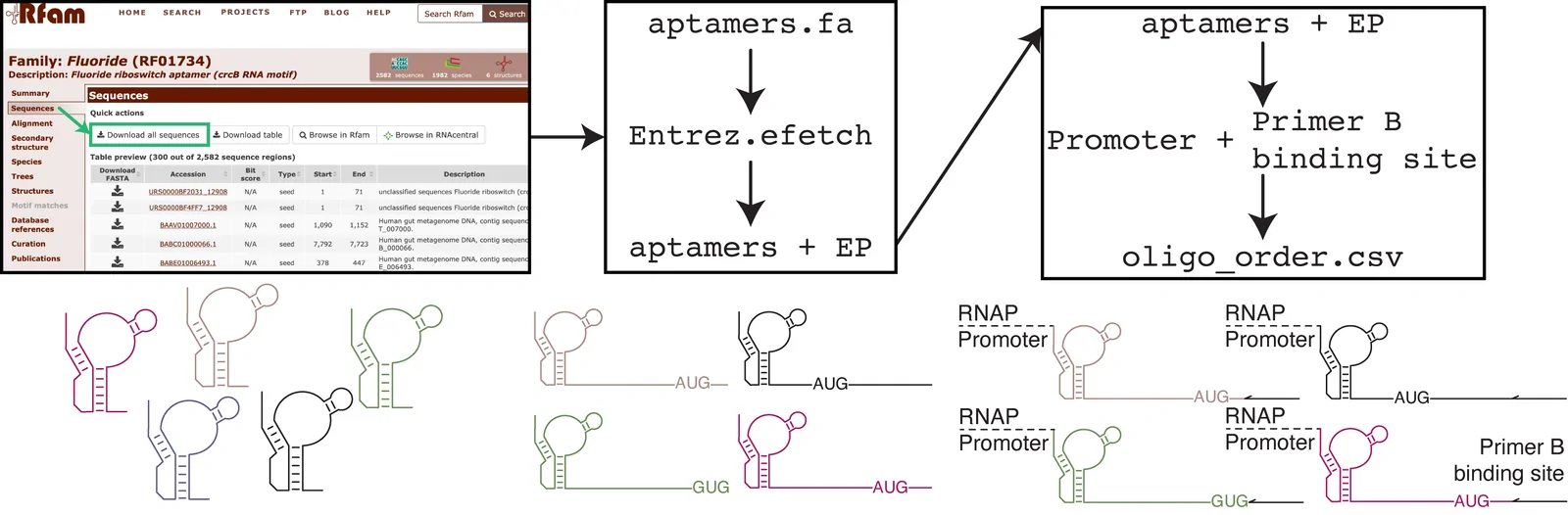
Visual schematic of the bioinformatic pipeline to generate complete riboswitches. A visual overview of acquiring the aptamer sequences from Rfam (step A1), then using the subsequent sequence information to locate the downstream sequence on the NCBI database (steps A3–6), and the ultimate oligo pool order (step A7). This figure is adapted from Hertz et al. (2026) *NAR* [1].

**A. Generate a riboswitch oligo pool (Figure 1)**

*Note: The following procedure is specifically for the fluoride riboswitch, but it can be replicated for any riboswitch aptamers listed on Rfam.*

**Figure 2.**
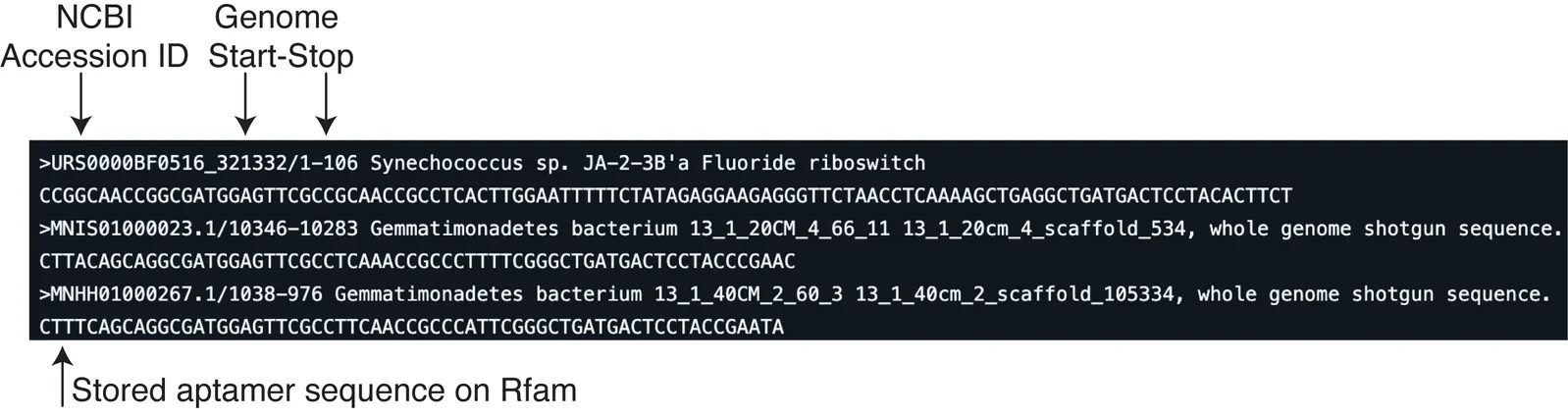
FASTA file head from Rfam. Rfam FASTA file provides the NCBI accession ID followed by the genomic position for the subsequent aptamer sequence on the next line. Notably, Rfam may also store synthetic sequences, e.g., the first FASTA entry in the figure; it typically starts with a >U, and the first genomic position is “1.” When conducting the aptamer extension, these entries must be processed out before calling the NCBI, or else, the code will receive an error.

1. Download all riboswitch aptamers sequences from the Sequence tab of the Rfam entry page (e.g., https://rfam.org/family/RF01734#tabview=tab1) [12] as a FASTA file (Figure 2).

2. Move the downloaded file to your working directory.

3. Create a data frame with columns for the NCBI accession ID, genome start, genome stop, and Rfam aptamer sequence.

4. For each sequence, remove the first 5 nts of the leading sequence that could impact riboswitch function.

*Note: Rfam may add a 5–10-nt buffer sequence at the 5′ and 3′ ends of the aptamer. The nts before the aptamer can impact riboswitch function [19,20]. This step intends to reduce potential interactions between the 5′ leader sequence and the aptamer.*

5. For each entry, extend the aptamer (variable name = stop) according to the DNA oligo synthesis limit.

*Notes:*

*1. For synthesizing an oligo pool from Twist Biosciences, the synthesis of oligos was capped at 300 nts. Thus, we used the following line to calculate the extended length for each sequence:*

extend_length = TWIST_max_nt_synthsis - (len(five_seq) + len(three_seq) + aptamer_length) - 1, *where* five_seq *is the RNA polymerase promoter region and* three_seq *is the 3′ primer binding site for oligo amplification.*

*2. If there is no DNA oligo synthesis limit, then identify the nt distance between the aptamer and the start codon. If the goal is translational riboswitches, then also add 15 nts for the first five codons.*

6. Submit each entry with ID, genome start, and calculated stop (step A5) to the NCBI via Entrez and retrieve the corresponding sequence.

*Note: This is automated with the Python Bio package and Entrez software (Entrez Programming Utilities Help, available at* *https://www.ncbi.nlm.nih.gov/books/NBK25501/* *). For the full code, please refer to the code script Bioinformatics_Covariation/Prepping_sequences.ipynb from the GitHub Code S1. For easy adaptation, the key code line is:*

handle = Entrez.efetch(db="nucleotide", id=accession, rettype="gb”, retmode="text", seq_start=start, seq_stop=stop)

7. To each sequence, append the *Escherichia coli* J23119 consensus RNA polymerase promoter sequence (from the registry of standardized biological parts, https://parts.igem.org/Part:BBa_J23119) to the 5' end and append a reverse primer binding site sequence to the 3′ end.

8. Export the sequences as a .csv file and order the ssDNA oligo pool.

*Note: Oligo pool synthesis biases toward shorter sequences. Ensure that oligos are close in length.*

9. Export the sequences as a FASTA file **with the promoter region removed** (e.g., “oligo_pool.fasta”) to create a synthetic genome of the **RNA transcripts** in Data analysis step A2.

**B. Generate a dsDNA riboswitch library**

1. Amplify the ssDNA oligo pool into the dsDNA template for the transcription reaction with the following PCR reaction (Table 1).

**Table 1.** Oligo pool amplification

Reagent	Final concentration	Volume
RNase/DNase-free water	n/a	15.75 μL
Oligo pool	0.4 ng/μL	0.5 μL
dNTPs	200 μM	0.5 μL
5× Q5 reaction buffer	1×	5 μL
Primer A	600 nM	1.5 μL
Primer B	600 nM	1.5 μL
Q5 polymerase	0.02 U/μL	0.25 μL
Total		25 μL

2. Run the following thermocycler protocol:

a. 95 °C | 3:00

b. 98 °C | 0:20

c. 53 °C | 0:15

d. 72 °C | 0:15 (*Note: Ensure this elongation time is compatible with oligo size.*)

e. Repeat steps b–d 14×

f. 72 °C | 2:00

g. 12 °C | Hold

3. Remove the remaining primers via bead purification according to the manufacturer’s protocol as follows:

a. Add 2.5× Cytiva Sera-Mag Select beads to each sample.

b. Vortex samples for 30 s and incubate at room temperature for 5 min.

c. Place samples on the magnetic stand and proceed with bead separation for 5 min.

d. Prepare fresh 85% ethanol.

e. Remove the supernatant from the beads.

f. Wash twice with 200 μL of fresh 85% ethanol.

g. Air-dry the beads on a magnetic rack for 5 min.

h. Remove tubes from the magnetic stand.

i. Elute in 50 μL of water.

j. Vortex for 30 s.

k. Incubate for 5 min.

l. Place the samples on a magnetic stand and proceed with bead separation for 5 min.

m. Aspirate the supernatant and place it in a new tube.

4. Quantify the purified dsDNA template using Qubit (high sensitivity).

5. Check the template purity through denaturing PAGE or bioAnalyzer.

6. Repeat steps B1–4 until enough dsDNA template is generated for all transcription reactions.

*Example: Two replicates of four transcription reactions, each with 100 nM of DNA, require at least 800 nM of dsDNA template.*

**Figure 3.**
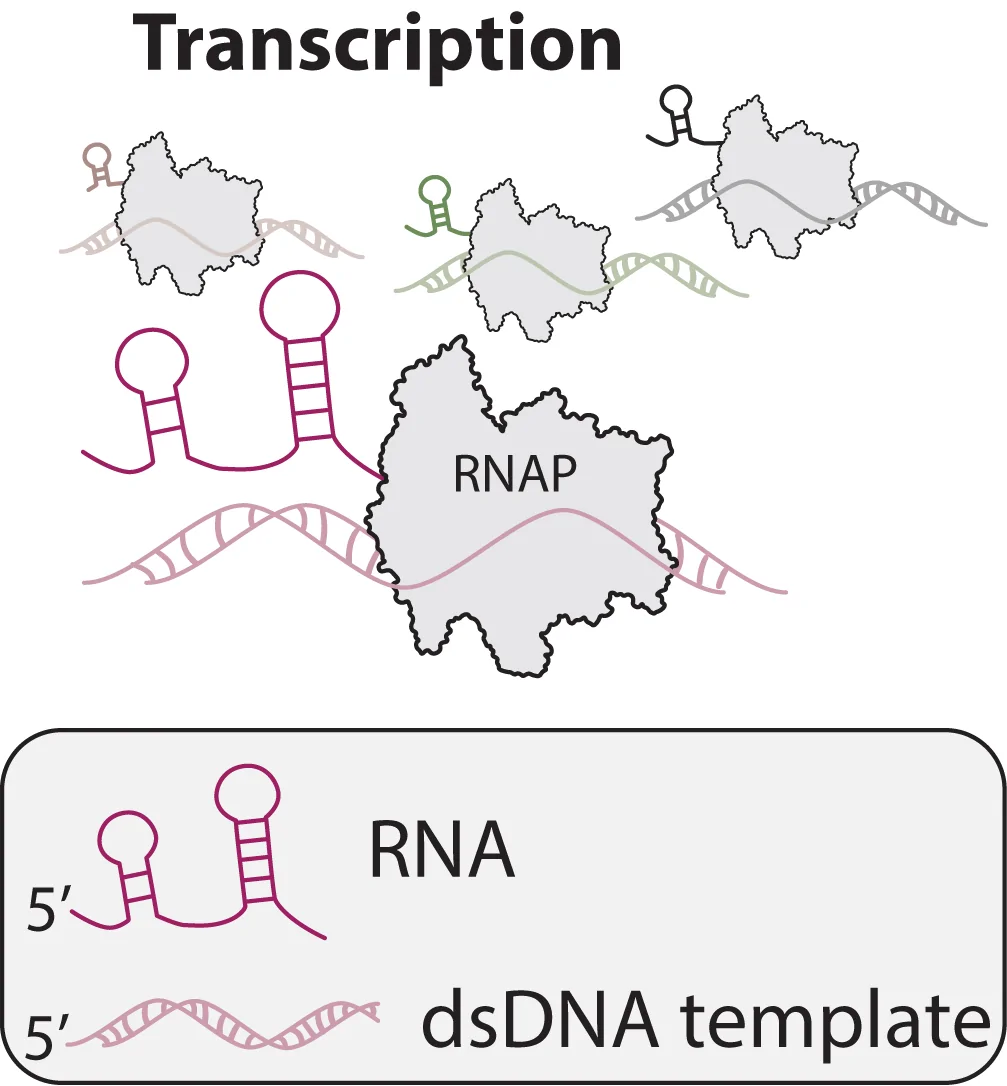
Visual schematic of a transcription reaction. RNAP, RNA polymerase; PDB ID: 7YPA. This figure is adapted from Hertz et al. (2026) *NAR* [1].

**C. Transcribe the dsDNA riboswitch library (Figure 3)**

1. In 0.7 mL tubes, add DNA, ligand condition, and water according to the experimental design.

*Note: See Protocol Setup S1 for a template for organizing the transcription reaction setups.*

2. To generate the transcription reaction (Table 2), follow these steps:

a. In 1.5 mL tubes, set up the 10× transcription buffer, transcription reaction master mix, and transcription start/stop solution.

b. To each 0.7 mL reaction tube, add the transcription reaction master mix.

c. Mix the reaction by pipetting up and down; then, place the reaction on ice.

d. Add 2 μL of *E. coli* RNAP holoenzyme to each reaction and place back on ice.

**Table 2.** Transcription reaction

Reagent	Final concentration	Volume
RNase/DNase-free water	n/a	12.75 μL – X μL
10× transcription buffer	1×	2.5 μL
MgCl_2_	5 mM	2.5 μL
BSA	1%	0.25 μL
Riboswitch ligand (e.g., NaCl or NaF)	10 mM	2.5 μL
DNA	100 nM	X μL
*E. coli* RNAP holoenzyme	2 units	2 μL
Total		22.5 μL

*Note: Master mix removes enzyme and DNA.*

3. Incubate the reactions at 37 °C for 10 min to form the transcription open complex.

*Note: Stagger the reaction tubes for easy downstream handling, e.g., tube A at 0:00, tube B at 0:30, tube C at 1:00, …*

4. At the 10-min mark, pipette 2.5 μL of the transcription start/stop solution and mix by pipetting up and down three times to ensure proper, quick mixing.

*Note: Following the staggered setup means adding the transcription start/stop solution to tube A at 10:00, tube B at 10:30, tube C at 11:00, …*

5. Incubate the reaction at 37 °C for 5 min.

*Note: Transcription reaction times vary depending on template content and length.*

6. Add 75 μL of TRIzol to end the reaction.

*Note: Following the staggered setup means adding TRIzol to tube A at 15:00, tube B at 15:30, tube C at 16:00, …*

7. Add 20 μL of chloroform to each reaction.

8. Vortex each reaction.

9. Incubate at room temperature for 2 min.

10. Centrifuge at 12,000× *g* for 5 min at 4 °C.

11. Transfer the top aqueous layer into a new 0.7 mL tube.

12. To each reaction, add, in order, 7 μL of 1 M NaCl, 1 μL of GlycoBlue, and 50 μL of isopropanol.

13. Incubate at room temperature for 10 min.

14. Centrifuge at 4 °C for 10 min at 21,130× *g* (max speed) with the tube hinge pointing upward.

15. Aspirate the supernatant, taking care not to disturb the (blue) pellet.

16. Wash the tube with 400 μL of ice-cold 70% ethanol.

17. Re-spin at 4 °C for 1 min at 21,130× *g* (max speed).

18. Remove the supernatant.

19. Air-dry the pellet for 5 min at room temperature with the cap open.

**D. RNA-seq of the transcribed riboswitches**

**D1. DNase digestion**

1. Resuspend the pellet in 43 μL of water.

2. Add 5 μL of 10× TURBO DNase buffer and 2 μL of TURBO DNase to each tube.

3. Incubate the reactions at 37 °C for 1 h.

4. Repeat steps C9–22, doubling the amount of TRIzol, chloroform, NaCl, and isopropanol.

**Figure 4.**
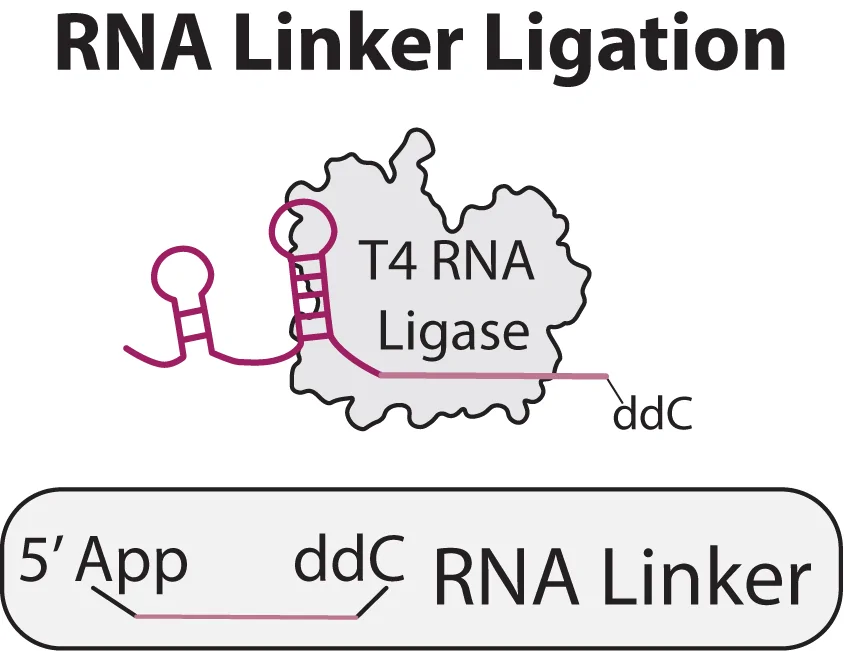
Visual schematic of the RNA linker ligation reaction. Ligation reaction of the 3' of the transcribed RNA to a known RNA linker sequence. PDB ID: 1S68. This figure is adapted from Hertz et al. (2026) *NAR* [1].

**D2. RNA linker ligation (Figure 4)**

1. Resuspend the pellet in 9 μL of water.

2. Make the linker ligation (Table 3), vortexing the master mix prior to adding enzymes.

*Note: See General note 3.*

**Table 3.** Linker ligation

Reagent	Final concentration	Volume
RNA sample	n/a	9 μL
50% PEG800	15%	6 μL
5′ App linker oligo	20 nM	2 μL
10× T4 RNA ligase buffer	1×	2 μL
T4 RNA ligase, Trunc KQ	5 units	0.5 μL
SUPERase·In^TM^ RNase inhibitor	10 units	0.5 μL
Total		20 μL

*Note: Linker is adenylated following the manufacturer’s protocol: NEB, M2610S.*

3. Add 11 μL of the master mix to each sample.

4. Incubate the reactions at 25 °C for 2 h.

5. To each reaction, add, in order, 130 μL of water, 15 μL of 3 M NaOAc, 1 μL of GlycoBlue, and 450 μL of ice-cold ethanol.

*Note: The water dilutes the PEG for an efficient precipitation reaction.*

6. Invert 3× and briefly spin down.

7. Store at -20 °C overnight or continue on after incubating at -80 °C for 30 min.

8. Centrifuge at 21,130× *g* (max speed) for 30 min at 4 °C.

9. Aspirate the supernatant, taking care not to disturb the pellet.

10. Wash the tube with 400 μL of ice-cold 70% ethanol.

11. Re-spin at 4 °C for 1 min at 21,130× *g* (max speed).

12. Remove the supernatant.

13. Air-dry the pellet for 5 min at room temperature with the cap open.

**Figure 5.**
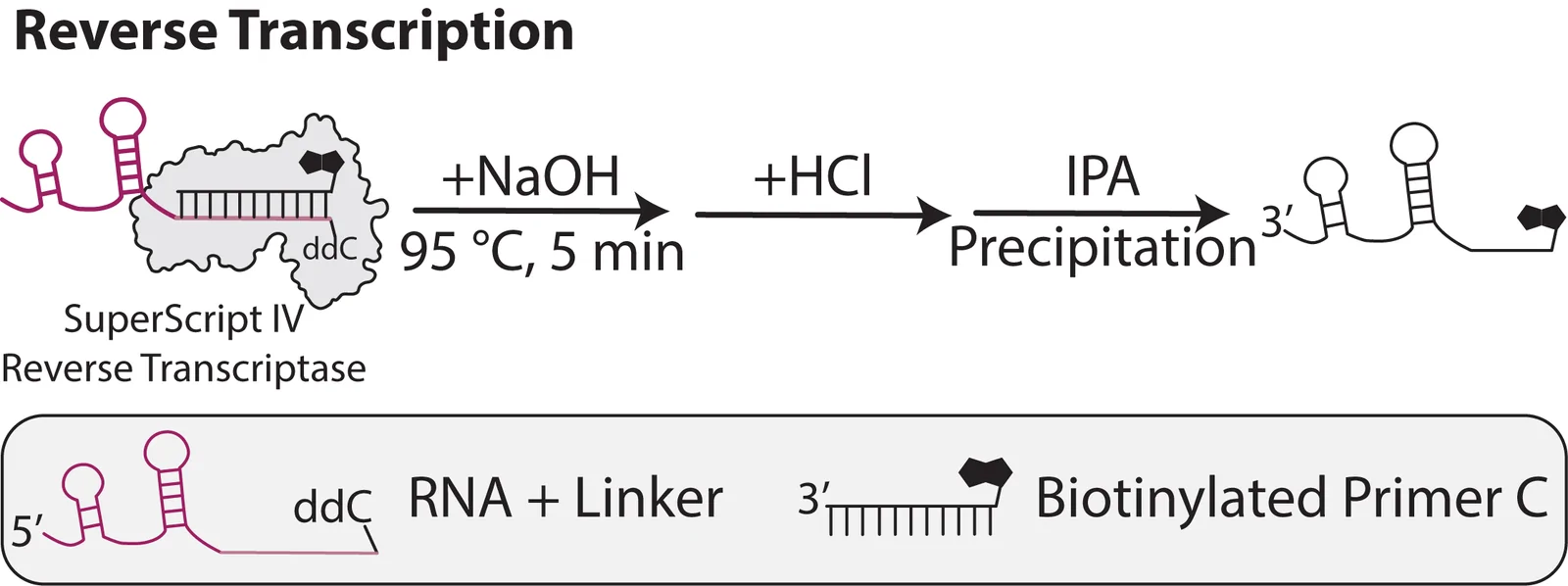
Visual schematic of the reverse transcription reaction and subsequent hydrolysis of the RNA. Primer C has a 5′ biotin modification to prevent 5′ ligation in section D4. PDB ID: 1MML. This figure is adapted from Hertz et al. (2026) *NAR* [1]. IPA, isopropanol.

**D3. Reverse transcription (Figure 5)**

1. Resuspend the RNA pellet in 3 μL of water.

2. Add 3 μL of 100 nM Primer C.

*Note: See General note 3.*

3. Place samples on the thermocycler and run as follows:

a. 95 °C | 2:00

b. 65 °C | 5:00

c. 25 °C | Forever

4. Prepare the master mix of the reverse transcription reaction (Table 4) without the RNA + Primer C.

**Table 4.** Reverse transcription

Reagent	Final concentration	Volume
RNA + Primer C	RNA: n/a; Primer C: 15 nM (0.3 pmol)	6 μL
RNase/DNase-free water	n/a	7.5 μL
5× SSIV buffer	1×	4 μL
dNTPs	500 μM	1 μL
DTT	5 mM	1 μL
SSIV	10 units	0.5 μL
Total		20 μL

5. At 25 °C, add 14 μL of the reverse transcription master mix to each sample and mix by pipetting gently.

6. Advance the thermocycler to the next steps:

a. 25 °C | 2:00

b. 50 °C | 10:00

c. 80 °C | 10:00

d. 4 °C | Forever

7. After at least 1 min at 4 °C, hydrolyze the RNA by adding 1 μL of 4 M NaOH.

8. Advance the thermocycler to the next step:

a. 95 °C | 5:00

b. 4 °C | Forever

9. After at least 1 min at 4 °C, remove the samples from the thermocycler to room temperature.

10. Add 2 μL of 1 M HCl and mix by pipetting.

11. Add 17 μL of isopropanol.

12. Incubate tubes at room temperature for 10 min.

13. Centrifuge at 21,130× *g* (max speed) for 10 min at 4 °C.

14. Aspirate the supernatant, taking care not to disturb the pellet.

15. Wash the tube with 400 μL of ice-cold 70% ethanol.

16. Re-spin at 4 °C for 1 min at 21,130× *g* (max speed).

17. Remove supernatant.

18. Air-dry each pellet for 5 min at room temperature with the cap open.

**Figure 6.**
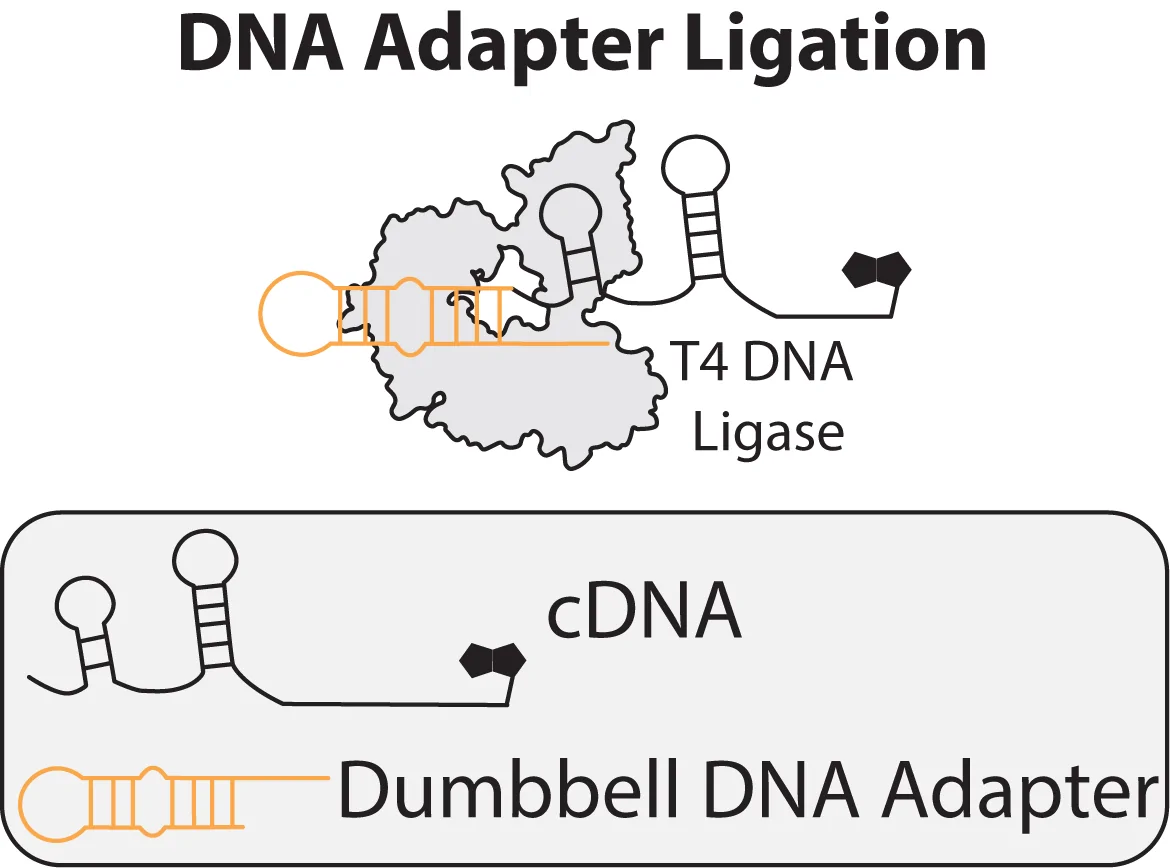
Visual schematic of the DNA ligation reaction. Ligation reaction of the 3′ end of the copyDNA product from reverse transcription to a DNA Adapter for Illumina Primers [21]. PDB ID: 6DT1. This figure is adapted from Hertz et al. (2026) *NAR* [1].

**D4. DNA adapter ligation (Figure 6)**

1. Resuspend each cDNA pellet in 7 μL of water.

2. Add 0.5 μL of 100 μM SS2.0 Dumbbell [21] to each reaction.

*Notes:*

*1. The final concentration of SS2.0 Dumbbell needs to be in great excess for efficient ligation.*

*2. Consider adding a “reference” tube of Dumbbell + Primer C without cDNA to use as a guide with downstream gel extraction. See General note 1.*

3. Place samples on the thermocycler and run:

a. 95 °C | 2:00

b. 21 °C | 3:00

4. Prepare the DNA ligation master mix according to the dumbbell ligation recipe (Table 5) without the cDNA and SS2.0 Dumbbell.

**Table 5.** Dumbbell ligation

Reagent	Final concentration	Volume
cDNA + SS2.0 Dumbbell	cDNA: n/a; Dumbbell: 2 µM	7.5 μL
50% PEG800	20%	10 μL
Betaine	500 mM	2.5 μL
10× T4 DNA ligase buffer	1×	2.5 μL
T4 DNA ligase	1,000 units	2.5 μL
Total		25 μL

5. Advance the thermocycler to the next step:

a. 30 °C | 2:00:00

b. 65 °C | 0:15:00

c. 4 °C | Forever

5. To each reaction, add, in order, 125 μL of water, 15 μL of 3 M NaOAc, 1 μL of GlycoBlue, and 450 μL of ice-cold ethanol.

6. Repeat steps D4.6–13 from the RNA linker ligation process.

7. Remove the excess SS2.0 Dumbbell via bead purification according to the manufacturer’s protocol. Aspirate the ssDNA samples with 20 μL of water.

*Note: See protocol details in step B3.*

**Figure 7.**
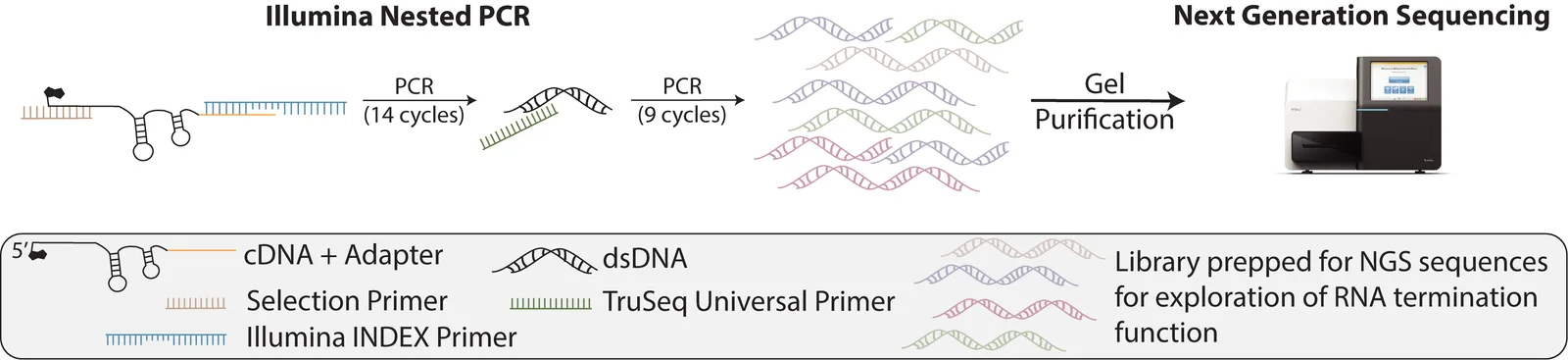
Visual schematic of the Illumina nested PCR workflow. This figure is adapted from Hertz et al. (2026) *NAR* [1].

**D5. Illumina nested PCR (Figure 7)**

1. Take 5 μL of ligated reaction and place it in a PCR tube.

2. To each tube, add 0.25 μL of 100 μM unique Illumina INDEX primer (Illumina Knowledge Article #7129).

3. Prepare the Illumina nested PCR master mix **without** the Illumina INDEX primer (added directly to the sample) and the TruSeq universal adapter primer (added during the PCR reaction) (Table 6).

**Table 6.** Illumina nested PCR

Reagent	Final concentration	Volume
ssDNA	n/a	5 μL
Illumina INDEX primer	500 nM	0.25 μL
RNase/DNase-free water	n/a	21 μL
5× Q5 reaction buffer	1×	10 μL
Primer D	25 nM	12.5 μL
dNTPs	100 μM	0.5 μL
Q5 polymerase	0.02 U/μL	0.5 μL
TruSeq universal adapter	500 nM	0.25 μL
Total		50 μL

4. Add 44.5 μL of master mix to each sample.

5. Place samples on the thermocycler and run:

a. 98 °C | 0:30

b. 98 °C | 0:10

c. 65 °C | 0:30

d. 72 °C | 0:30

e. Repeat steps b–d 14×

f. 12 °C | Forever

6. Add 0.25 μL of 100 μM TruSeq universal adapter primer to each tube.

7. Advance the thermocycler to the next step:

a. 98 °C | 0:10

b. 65 °C | 0:30

c. 72 °C | 0:30

d. Repeat steps a–c 9×

e. 72 °C | 5:00

f. 4 °C | 3:00

g. 4 °C | Forever

8. Add 0.25 μL of ExoI to each tube.

9. Advance the thermocycler to the next step:

1) 37 °C | 30:00

2) 80 °C | 20:00

3) 4 °C | Forever

10. Perform silicon column-based PCR clean up according to the manufacturer’s protocol as follows:

a. Add 5× buffer PB to the sample.

b. Add the sample to a silicon column on a vacuum system.

c. Apply vacuum.

d. Wash 2× with 750 μL of buffer PE.

e. Transfer the column into the collection tube.

f. Dry spin in a centrifuge at 21,130× *g* (max speed) for 1 min.

g. Elute the PCR product in 15 μL by centrifuging at 21,130× *g* (max speed) for 1 min.

11. Remove excess primers through gel purification on an 8% urea PAGE gel.

12. (Optional) Run 1 μL of post-gel-purified product on another 8% urea PAGE gel for quality control.

13. Send the dsDNA sequencing library to the NGS core facility for a paired-end 150-cycle sequencing.

## Data analysis

**A. Genome assembly**

*Note: The following was done on Northwestern’s high-performance computing system running Red Hat Enterprise Linux.*

1. In the desired working directory, load megahit [17].

$ module load megahit/1.0.6.1

2. Using the FASTA file created in Procedure step A9, generate a genome assembly:

$ megahit -o megahit_OligoPool_genome_directory -r oligo_pool.fasta

3. To calibrate the genome assembly, use the STAR genomeGenerate mode [18]:

$ module load STAR/2.7.9a

$ STAR --runMode genomeGenerate --genomeDir megahit_OligoPool_genome_directory --genomeFastaFiles oligo_pool.fasta --genomeSAindexNbases 8

**B. Assigning reads to bioinformatic information**

*Note: The following was done on Northwestern’s high-performance computing system running Red Hat Enterprise Linux.*

**Figure 8.**
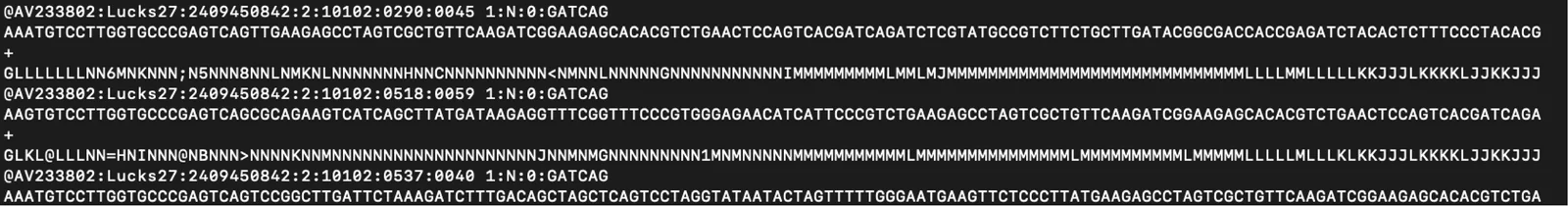
FASTQ file architecture. The first 10 lines of the raw .fastq.gz file.

1. In the directory with the NGS .gz output files (Figure 8), compile PEAR according to author guidelines (https://github.com/tseemann/PEAR?tab=readme-ov-file) to combine the paired-end reads [16]:

$ conda activate pear-env

*Note: pear-env is described on this project’s GitHub.*

$ pear -f NGS_R1.fastq.gz -r NGS_R2.fastq.gz -o NGS/NGS_PEAR -y 4G -j 1

$ conda deactivate

2. Change wd to NGS and write a bash file to run the following STAR script:

STAR --runMode alignReads \

--alignIntronMax 1 \

--alignEndsType Local \

--alignSoftClipAtReferenceEnds Yes \

--outFilterScoreMinOverLread 0.5 \

--outFilterMatchNminOverLread 0.5 \

--outSAMmultNmax 1

*Note: For additional context on the parameters, please refer to the supplemental document “STAR_Documentation.pdf.”*

**Figure 9.**
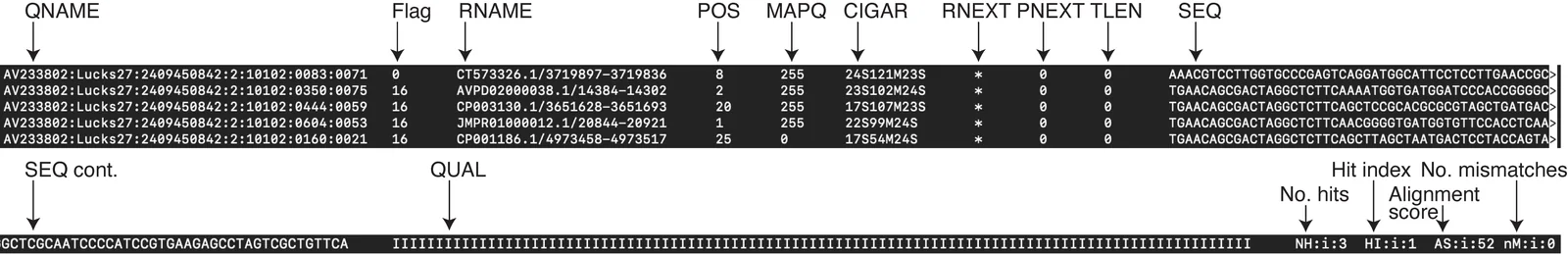
SAM file architecture. The bottom line is a right-horizontal continuation of a line from the top section. Documentation about SAM files can be found at https://samtools.github.io/hts-specs/SAMv1.pdf.

3. The output will be a sequence alignment/map (SAM) file (Figure 9):

**C. Calculating percent termination as a measure of riboswitch functionality**

*Note: The following was done on Northwestern’s high-performance computing analytics nodes running Jupyter.*

**Figure 10.**
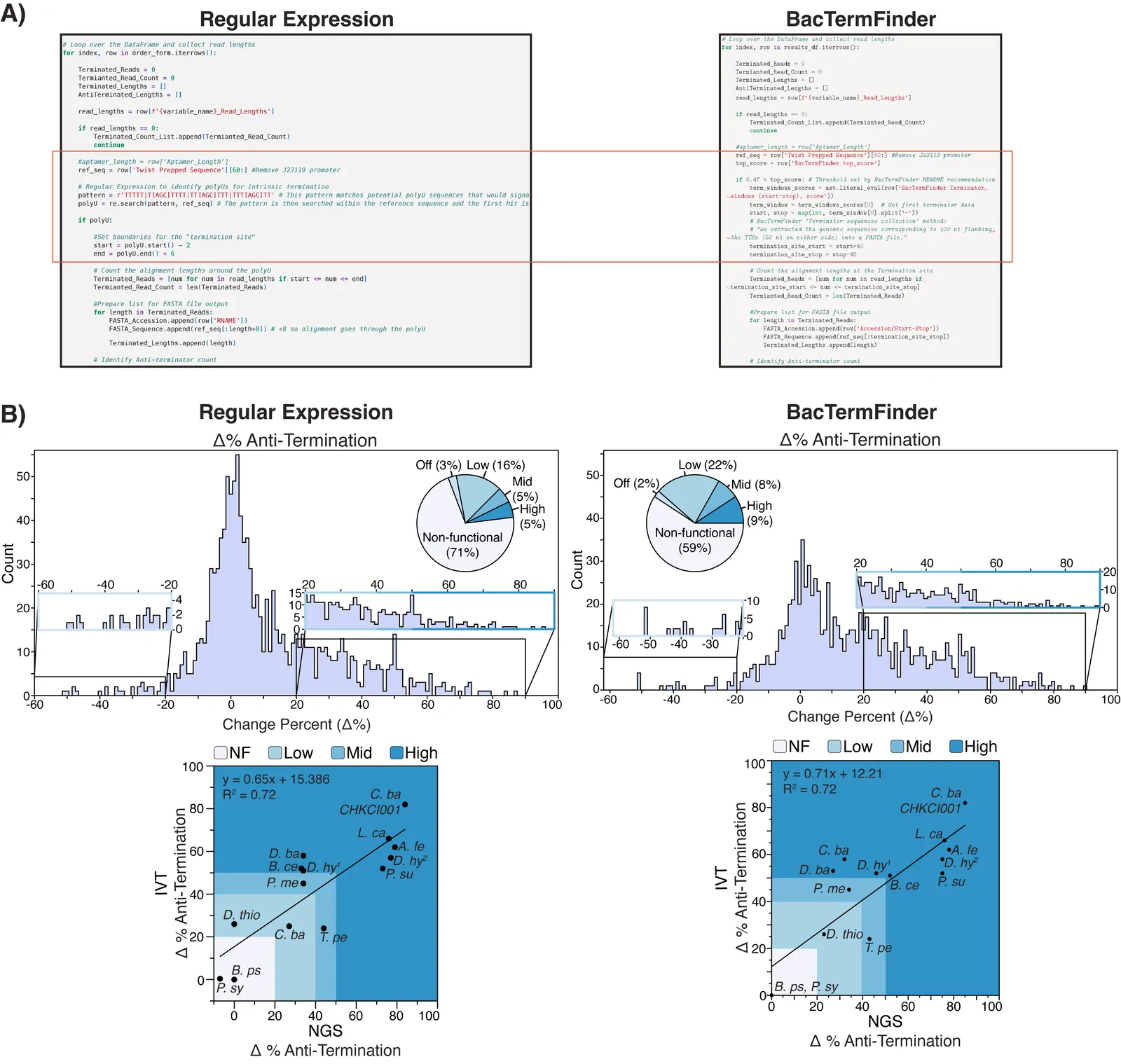
Comparison of two termination site detection methods. (A) Side-by-side of the code to determine termination sites and the reads that fall within that range, with the regular expression on the left and the BacTermFinder on the right. Both are available in Code S1. (B) Distribution of riboswitch functionality analysis determined by termination change of (anti-termination % with fluoride) – (anti-termination % no fluoride) (top). Correlation plots to individual validation of riboswitch functionality (bottom). Regular expression results on the left and the BacTermFinder results on the right. This figure is adapted from Hertz et al. (2026) *NAR* [1].

1. Determine the method for identifying termination sites: regular expression or BacTermFinder [15] (Figure 10A).

*Note: BacTermFinder is computationally intensive. If running it is not possible, we found that using the following regular expression pattern yields similar results (Figure 10).*

a. Regular expression, re.search(pattern, read) to identify the first match:

pattern = r'TTTTT|T[AGC]TTTT|TT[AGC]TTT|TTT[AGC]TT'

*Note: The termination window is defined to fit around the RNAP footprint and account for experimental stochasticity with 2 nt upstream the polyU and 6 nt downstream the end of the polyU.*

b. BacTermFinder:

i. Set up the system on a Linux processor according to BacTermFinder GitHub (https://github.com/BioinformaticsLabAtMUN/BacTermFinder).

ii. Write a bash file to run the following script in a Linux system:

$ python genome_scan.py oligo_pool.fasta [Sliding window step] [Prefix for output files] [Feature generation batch size] > log.out

*Note: As per the guidance of the BacTermFinder GitHub:*

*Sliding window step = 3*

*Prefix for output files = out*

*Feature generation batch size = 10000*

iii. Using Python, organize the out*.fasta_mean.csv file to combine the terminator windows for each variant, e.g., from GitHub Code S1: BacTermFinder_Processing.ipynb. Export the data frame to a new .csv.

2. Using Python, position the reads to their respective position in their aligned variant sequence. Then, save each read’s aligned end position for each variant. Loop through each variant and count the reads that fall within the termination window (determined either through regular expression or BacTermFinder). Export the processed data to a new .csv.

*Note: Read_Classification_[METHOD].ipynb from the GitHub Code S1.*

3. Export the spreadsheet for analysis according to the experimental design, e.g., Figure 10B.

## Validation of protocol

There should be at least two replicates for each experimental condition.

Original data files and NGS output samples can be found on the cited GitHub.

This protocol has been used and validated in the following research article(s):

• Hertz et al. [1]. High-throughput functional profiling and evolutionary covariation analysis of entire riboswitch sequences. *NAR*, 2026-02. https://doi.org/10.1093/nar/gkag542

PEAR merged rate: 30%–80%.

STAR mapping rate: 50%–60% (while below the recommended rate, likely due to incomplete removal of RTprimer-Dumbbell from the library).

Read-depth threshold per riboswitch: >10 total reads (terminated + anti-terminated).

Replicate correlation: The Pearson correlation coefficient between replicates should be >0.97.

QC pass/fail criteria: The aligned mapped read had to start within the first 10 nts of the riboswitch variant sequence, go beyond the aptamer sequence, and be the first hit/highest alignment score (anticipated with outSAMmultNmax parameter).

## General notes and troubleshooting

1. Shorter fragments are more likely to bind the NGS flow cell (Illumina Knowledge Article #3874). If possible, design oligo pool sequences such that the full-length transcripts are not much longer than anticipated terminated lengths.

2. Oligo pool synthesis biases shorter oligo sequences. Ensure that all oligo sequences are similar in length.

3. Primer and oligo concentrations, except for the Dumbbell, can be altered if desired. We suggest performing the biochemical reaction with a range of oligo concentrations and running a PAGE gel to check reaction efficiency.

4. Results from this protocol are limited to the RNA polymerase used in the transcription. For example, while *C. ba CHKCI001* was the highest performer when transcribed by the *E. coli* RNA polymerase (Figure 10B), the same functionality for *C. ba CHKCI001* cannot be assumed with its own RNA polymerase in its native cell environment.

5. If there are issues with yield after the ethanol precipitation steps post-ligations (Procedure steps D2.5 and D4.6), then consider diluting the PEG-containing reaction with more water and adjusting the ratios of ethanol and NaOAc.

6. Beads will not remove all Dumbbell, which can be ligated to RT primer and lead to short side products that will end up in the final library without a gel purification step. Gel purification post-dumbbell and other steps can be considered, as done elsewhere [11].

7. When adapting this protocol, it is recommended to run a PAGE gel post-chemical reactions (ligations, RT, and PCRs) to check reaction quality.

## Supplementary information

The following supporting information can be downloaded here:

1. Code S1. Project GitHub: https://github.com/LucksLab/Hertz_HighTroughput_Riboswitch_Discovery_2025/tree/main

2. Protocol Setup S1. Supplemental_Transcription_Template.xlsx

3. STAR parameters explained: STAR_Documentation.pdf
